# Iranian midwives’ lived experiences of providing continuous midwife-led intrapartum care: a qualitative study

**DOI:** 10.1186/s12884-022-05040-z

**Published:** 2022-09-23

**Authors:** Leila Amiri-Farahani, Maryam Gharacheh, Narges Sadeghzadeh, Hamid Peyravi, Sally Pezaro

**Affiliations:** 1 Nursing and Midwifery Care Research Center, Department of Reproductive Health and Midwifery, School of Nursing and Midwifery, Iran University of Medical Sciences, Tehran, 1996713883 Iran; 2 Nursing and Midwifery Care Research Center, School of Nursing and Midwifery, Iran University of Medical Sciences, Tehran, Iran; 3 The Centre for Healthcare research, Coventry University, Coventry, UK

**Keywords:** Midwives, Midwifery care, Woman-centered care, Qualitative study, Midwife-led

## Abstract

**Background & Objective:**

Continuity of midwifery-led care during labour and birth is considered optimal. To ensure its sustainability in practice where limited evidence is available, the aim of the present study was to explore midwives’ lived experiences of delivering continuous midwife-led intrapartum care.

**Methods:**

This study took a qualitative approach in meeting its aim. Participants were midwives working in the labour wards of private and public hospitals in Iran. The data were purposefully collected in 2019 through in-depth, semi-structured, and face-to-face interviews with midwives (*n* = 10) aged between 26 and 55 years. A thematic analysis based on descriptive phenomenology was undertaken to make sense of the data collected.

**Results:**

“Wanting to lead continuous woman-centered care but being unable to” was identified as an overarching theme. Three other themes “emphasis on the non-interventional care”, “midwifery-specific focus” and “barriers and challenges of midwifery care” were also identified. Ultimately, midwives described knowing how to and wanting to lead continuous ‘woman’-centered care but being unable to. Perceived barriers included lack of familiarity with and knowledge in relation to childbirth, the insignificant role of midwives in decision making, obstetrician utilitarianism, high workloads along with work-related stress argument-driven communication between midwives and obstetricians and an absence of a ‘triangle of trust’ in care.

**Conclusion:**

Future research strategies could usefully include obstetricians and focus on the upscaling of midwifery in Iran using continuity of care models, highlight the value of midwives, identify why uptake of antenatal education in Iran is poor and develop user friendly, evidence based, midwife-led programs. Initiatives aiming to promote mutual professional respect, trust and collegiality and increased remuneration for midwifery work would be also welcomed in pursuit of reducing maternal and infant mortality in Iran.

## Introduction

In both midwifery and nursing practice, ‘care’ is a core component [1], and an essential communicative action [2]. In midwifery, the concept of care is frequently associated with being “with woman”, though we recognise that not all people who birth will identify themselves as such. In this sense, the midwife, as a constant companion, creates a trustful relationship with birthing women and people through the rebalancing of power, division of responsibilities and mutual agreement [3]. In Hunter’s studies, “being with woman” is translated to mean that the midwife provides physical, mental, psychological, and emotional support according to the desires of women in labour, increasing mutual trust [4]. Importantly, the findings of both quantitative and qualitative studies demonstrate that those who receive such care continually enjoy several psychological and physiological advantages such as needing less pain relief during labour and shorter spontaneous labours, as well as greater satisfaction with their childbirth experiences [5, 6].

The World Health Organization (WHO) has expanded the definition of “Respectful Maternity Care” in order to improve clinical practice and to protect the health of mothers and newborns, and to eliminate discrimination and provide a fair and effective health system [7]. It has issued a statement on disrespect and abuse (D&A) during childbirth, which emphasizes the importance of respectful care of the mother, protecting her rights during pregnancy and childbirth, and the need for urgent attention to this global phenomenon [8]. The importance of the birth experience is so great that the WHO, while emphasizing the health of the mother and child and promoting the mother’s mental health, has considered strategies to improve the birth experience, which include: increasing support for respectful care and implementing interventions to reduce disrespect and abuse during care in labour [8]. The WHO considers good interaction as a prerequisite for positive birth outcomes; this interaction is maintaining respect, providing adequate information and emotional support during pregnancy and childbirth [9].

Continuous support delivered during labour is also an essential component of midwifery care from both the perspective of both those childbearing and midwives [10–12]. In this regard, participants in Lundgren’s study reported continuous communication with the midwife during labour, leading to greater self-esteem and greater satisfaction [13]. Elsewhere, recipients of midwifery-led care referred to midwives positively as “being there” and midwives described “being present” [10].

Midwife led care can be described as being ‘woman-centered’, though we recognise that there may be some people who birth who do not identify as such. Woman-centered care is a philosophy and a consciously chosen tool for the care management of the childbearing woman, where the collaborative relationship between the woman - as an individual human being - and the midwife - as an individual and professional - is shaped through co-humanity and interaction; recognizing and respecting one another’s respective fields of expertise [14]. Woman-centered care has a dual and equal focus on the woman’s individual experience, meaning and manageability of childbearing and childbirth, as well as on health and wellbeing of mother and child. Woman-centered care has a reciprocal character but fluctuates in equality and locus of control [14].

Continuous care led by midwives is highly valued [15]. Essential components of this include professional knowledge, expertise, and sensitivity [4], attentiveness, education, support and guidance [16, 17], the giving of advice and information [4], along with mutual, flexible and trustful communication [18]. Such care can increase trust in one’s capacity to give birth [13]. Continuous, midwife-led during labour means being with the birthing woman at least during their active phase of labour [6]. Significantly, this leads to more positive health outcomes for the mother and the newborn, as well as fewer interventions during labour [19]. Nevertheless, whilst many studies have focused on patient satisfaction along with the consequences, safety, and effectiveness of this type of care, little is known about the individual lived experiences of the midwives delivering it. Such insights will be important to the sustainability of such midwifery care delivered in future. Consequently, the aim of the present study was to explore midwives’ lived experiences of delivering continuous midwife-led intrapartum care. Our aim subsequently informed our research question: What are the lived experiences of midwives delivering continuous midwife-led intrapartum care?

## Method

This study was undertaken using a qualitative descriptive approach and guided by the methodological principles of emphasizing openness, questioning pre-understanding, and adopting a reflective attitude [20]. A qualitative approach to data collection and analysis was considered most appropriate in meeting our studies aim as it enables researchers to discover and interpret subjective phenomena rooted in the lived experiences of individuals while emphasizing the need to pay attention to social and historical influences to explain hidden meanings [21].

Ethical approval for this study was obtained from the Ethics Committee of the Research Deputy in Iran University of Medical Sciences (code: IR.IUMS.REC 1396.0014). A written informed consent was obtained from participants prior to their participation and interview process. The participants were informed that they could leave the study at any time. They were also assured that every measure would be taken to maintain Privacy and confidentiality throughout the research including the use of pseudonyms to protect their identity.

Recruitment began once ethical approval had been granted. Midwives were invited to participate if they had at least 6 months experience in working on the labour ward, experience in delivering midwifery care, communication skills and the ability to share personal experiences with the researcher, and no history of mental disorders. Participants with maximum variation in terms of age, work experience, and type of hospital (public or private) were selected.

To gain participants’ lived experience of midwifery care during labour and birth, 12 in-depth and semi-structured interviews were carried out in-person with 10 experienced midwives. To gain the trust of midwives, long-term interactions with the research team was also encouraged. Prior to each interview, in addition to obtaining informed consent, permission to record the interview was also obtained from the participants, and they were given the right to leave the study at any time. Subsequently, participants were invited to complete the demographic characteristics questionnaire, the results of which are presented in Table 1.

**Table 1:** Demographic characteristic of participants

Pseudo name of participant	Age of participant	Participant education	Work experience (Years)	Marriage status	Type of hospital
Zahra	28	Bachelor	6	Single	Private
Fatemeh	31	MSc	6	Single	Private
Elmira	27	MSc	4	Single	Governmental
Fatima	26	MSc	3.5	Married	Private
Ashraf	45	Bachelor	15	Married	Governmental
Nadereh	55	Bachelor	30	Married	Governmental
Farahnaz	37	Bachelor	11.5	Married	Governmental
Mary	51	Bachelor	26	Married	Governmental
Parvaneh	26	Bachelor	2	Married	Governmental
Nafiseh	38	Bachelor	13	Married	Governmental

Interviews were conducted by L.A.F, a researcher with a PhD in reproductive health, 11 years of work experience in the labour ward as a midwife, and competence in conducting qualitative interviews. Interviews were conducted in a quiet room within the maternity department and in some cases, in the School of Nursing and Midwifery of Iran University of Medical Sciences with the participants’ consent. Interviews (*n* = 10) lasted between 45 and 120 minutes. Questions asked during interview included: 1) How does caring for a woman during childbirth make you feel? 2) Can you share your experience of 1 day caring for a woman during childbirth? 3) How is the care of a woman during labour? a) What is it like? b) What does it mean?

If necessary, follow-up questions such as: “Please explain more “and “What do you mean by that?” were used to garner clarification on the participants’ answers. After each interview, the researcher created field notes about the non-verbal communication of the interviewee as well as their own observations, which were used for reflection during analysis. The recorded interviews were then transcribed verbatim. MaxQDA-10 software was used to manage, record and store data.

A thematic analysis based on descriptive phenomenology was undertaken to make sense of the data [20]. In line with this approach, the goal of our analysis was to achieve an understanding of patterns of meanings from data on the lived experiences of our participants. Accordingly, the first author primarily achieved familiarity with the data through open-minded reading and re-reading to explore experiences whilst keeping the aim of our study in mind. Subsequently, meanings corresponding to our study aim were highlighted and related to each other to compare and identify differences and similarities. Thereafter, via an iterative succession of refinements in partnership with the wider research team, patterns of meanings were further identified and examined. Lastly, the research team organized these meanings into patterns and, finally, themes and subthemes. To aid this process, reflective discussions were held between the research team throughout, particularly as tentative themes were being identified from the data.

In line with our approach, we considered reflexivity, credibility, and transferability as concepts important to acknowledge throughout the research process to engender validity and rigor [20]. Lincoln & Guba’s criteria were used to evaluate the study rigor specifically [22]. To ensure confirmability, member-checking and peer-debriefing methods were used. In this sense, findings were reviewed by the participants for accuracy of interpretation [23]. An attempt was also made to increase the transferability of the findings by providing a detailed explanation of study steps and a clear description of themes.

## Results

A total of 10 midwives aged between 26 and 55 years with between two and 30 years of work experience participated in this study. The participants’ demographic characteristics are presented in Table 1. In the present study, the overarching theme extracted was midwives’ “Wanting to lead continuous woman-centered care but being unable to” (Table 2). In line with our chosen method, meanings found from participants experiences are described in text organized under themes and subthemes below, illustrated with quotes.

**Table 2:** The overarching theme, themes, sub-themes, and concepts that formed the participants’ lived experiences of maternal care during labour and delivery

Overarching theme	Theme	Sub-theme	Concepts
Wanting to lead continuous woman-centered care but being unable to	1.Emphasis on the non-interventional care	Emphasis on physiological childbirth	–
		Psychological care is more important than physical care	–
	2.Midwifery-specific focus	Midwife’s professional values	Justice in care
			Communication skills
			A sense of empathy
			Sense of responsibility and work ethics
			Professional view of work
		Midwife’s characteristics and competence in providing care	Patience
			An interest in the job
			Right reaction in critical situations
			Ability to do teamwork
			Ability to control stress
			Optimism
	3.Obstacles and challenges of midwifery care	Lack of familiarity with and knowledge in relation to childbirth	Pregnant woman’s unfamiliarity with the process of physiological childbirth
			Pregnant woman’s unfamiliarity with the complications of cesarean section
		The insignificant role of midwives in decision making	Midwife is in the margins of decision making
			Lack of midwife independence in the care process
			Robotic care
		Obstetrician utilitarianism	Performing all deliveries
			Not giving midwife the freedom to perform delivery
			Lack of teamwork
		High workloads along with work-related stress	Multiple responsibilities along with work-related stress
			Simultaneous responsibility of care for several women/people in labour
		Argument-driven communication between obstetricians and midwives	Argument over the diagnosis of problem
			Argument along with disrespect
			Argument over financial issues
		An absence of a ‘triangle of trust’ in care	Patient’s trust in midwife
			obstetrician’s trust in midwife

### Theme One: Emphasis on the non-interventional care

Within this theme, participants broadly described the benefits of being able to provide continuous midwifery-led care without medical intervention. Sub-themes included “emphasis on physiological childbirth” and the perception that “psychological care is more important than physical care”. One participant reflected how being able to provide this type of care may result in better outcomes and less unnecessary medical intervention in physiological childbirth: *“It means that you would be able to deliver care at the best time. For example, to a woman who has a dilatation of 3 cm, we usually say that she has entered the active phase and we should not tear the water bag at this time. If they allow the delivery to progress on its own, it will definitely have a better outcome. If a good care is delivered, it will make the patient receive less intervention, and the process will continue easier.”*

In addition to reflections on the provision of continuous midwifery-led care resulting in fewer unnecessary medical interventions, participants often considered that the provision of psychological care was of equal if not more importance than the provision of physical care in this context: *“When it comes to childbirth and how a midwife supports a woman in labour, I think it’s my job to be able to manage a good labour. Midwifery is not just being able to pull the baby out, but to provide psychological and physical support for woman in labour and to allow childbirth to be progress with less intervention and successfully managing the woman in labour.”*

### Theme Two: Midwifery-specific focus

This theme captures participant contributions which emphasize the importance of the midwife’s constant presence at the woman’s side. Two subthemes were also extracted in relation to the midwife’s professional values ​​and midwife’s personal characteristics and competence in providing care. Additional concepts associated with these subthemes are presented in Table 2.

In this theme, participants broadly describe the professional values and personal characteristics of a midwife as being a combination of personality traits developed through life in relation to both past personal experiences and institutional experiences. Participants defined their own professional values in concepts such as embracing justice in care, strong communication skills, a sense of empathy, a sense of responsibility, a strong work ethics, and professional view of the job. These characteristics were reportedly interactive with individual characteristics such as patience, the ability to work in a team, quick reactions in critical situations, the ability to control stress, and having a positive attitude towards life.

One participant expressed their professional values ​​as being *“A sense of conscience [which] compels me to take care of the patient according to standard and basic principles. I try to do my job properly even when there is no supervision of an obstetrician.”*

Maryam described the need for competence in providing care, as *“The midwife must be able to control her own stress in the process of caring for patient. Our job is always full of stress and there are times that the life of woman and infant is at risk, and if you cannot control the situation well and overcome stress, you may not get a good result.”* She continued to reflect on how a midwife’s characteristics are integrated with their level of competence as *“It is very important what type of energy you radiate in space and whether you have a positive energy and attitude that everything will go well, or you are worried and waiting for bad things to happen. You will eventually get good results when you transfer positive energy to patient and environment.”*

### Theme Three: Obstacles and challenges of midwifery care

This theme captured a multitude of major challenges midwives face in the effective delivery of continuous midwifery-led care. These included encountering those in labour being unfamiliar with the process of physiological childbirth, high work pressure, work-related stress, and challenging communication with obstetricians. These challenges had devastating effects on almost every aspect of the participants’ lived experience of midwifery care (Table 2).

### Lack of familiarity with and knowledge in relation to childbirth

This sub-theme refers to one of the challenges of professional life of midwives who, in providing midwifery care, care for those who do not have adequate knowledge in relation to childbirth processes. Many are therefore entering an unknown world and are consequently afraid of childbirth and request elective cesarean delivery. For example, Zahra described her experience of childbirth being unknown phenomenon and stated: *“Almost all patients who come here [labour ward] are at the first time. They have a complaint, pain or bleeding when they enter the labour. They enter the labour a little anxious and scared, because they have not attended childbirth classes. Even though we offer childbirth classes in our hospital, these classes are not so active because people are not very enthusiastic about them.”* Zahra further reported that *“What we have been doing recently is explaining the complications of cesarean section such as hematoma and intestine adhesion. Patients usually do not listen to our advice at all and argue that they will not have vaginal delivery even if they die.”*

### The insignificant role of midwife in decision making

The insignificant role of midwives in decision-making captured within this subtheme refers to the lack of midwives’ autonomy in decision-making and the lack value placed upon them in practice. As an example, one participant described one such experience where *“We had a patient last night who had dilation of 7-8 cm and she was progressing well. Then the patient deteriorated and her O2 saturation dropped, so that we had to administer some oxygen, and obstetrician took her for cesarean section. We could not comment, because we have no role in decision making.”*

In relation to the lack of value placed upon midwives in practice, one participant narrated; *“I feel like I’m a robot in providing care. I feel like midwifery is a dependent profession. In a way, we have to do flattery to someone. Obstetrician gives order and we have to implement it, then the obstetrician comes and blames us for not doing certain things; I kind of feel that I am a robot.”*

### Obstetrician utilitarianism

Obstetrician utilitarianism is one of the obstacles and challenges of midwifery care captured within this subtheme that midwives are dealing with daily. In practice, obstetricians are observed to usurp the management of labour and intrapartum care. In some cases, participants purported that this is so that they did not have to share any income generated from intrapartum care with the midwives. Zahra outlined one such experience as follows: *“If patient has dilation of 6-7 centimeters, we have to call the obstetrician to come and perform the delivery. Usually, because of financial issues, the obstetricians perform all deliveries because they do not want to share their income. If they are very desperate, they will ask us to perform the delivery, but they administer the epidural in order not to give us too much money.”*

One participant described their experience of having the management of care usurped by an obstetrician and reflected; *“It disappoints me when I do my best to take care of a patient, but I see that the doctor does his best to perform delivery by herself and does not treat me as a person who has been there for hours taking care of patient.”*

### High workload along with work-related stress

Midwives describe in this subtheme how the high prevalence of work-related stress can negatively impact upon the quality of midwifery care provided. This is often resultant in the provision of physical rather than holistic care. For example, Fatemeh, in charge of the labour rooms, describes; *“Our workload has increased so much. Now every midwife is responsible for at least three patients. The midwife in charge of newborn must be next to mother to teach her how to breastfeed. Finally, no matter how good we are, we cannot provide good psychological support for patients during labour.”*

### Argument-driven communication between obstetrician and midwife

Another challenge of midwifery care described within this subtheme is the ague-driven communication between obstetricians and midwives. Interference in physiological childbirth and the care given during labour provides a context for controversy. Elmira for example reflected on such interactions and stated: *“If the thoughts of obstetrician and midwife are on the same page, there is usually no problem, but if there is a conflict of opinions, there will be argument.”*

Some of the disputes between the obstetrician and midwife are described as being related to resentment and financial issues. In these conflicts, some obstetricians were described as striving to provide all intrapartum care to secure higher income. For example, one participant reflected; *“I bear high level of stress during childbirth, worrying about placental abruption, fetal heart rate and hypoxia, and it is the obstetrician who gets paid for it. Why should I bear so much stress? The obstetrician does not say that part of income and benefits should go to the midwife. As a midwife, I say let it go, why should I have to endure so much stress?*

### An absence of a ‘triangle of trust’ in care

The need to form a triangle of trust in care where there is absence is one of the challenges commonly reflected on through participant statements captured within this subtheme. In this triangle is an obstetrician, a pregnant woman, and a midwife, where the obstetrician is seemingly always at the top of the hierarchy.

Regarding the relationship between the obstetrician and the midwife, there was also a distinct need identified to build on collegial trust in particular: *“Trust happens to be very effective. Doctors are very different. For instance, some of them do not tolerate stress at all, so they want to make decision quickly and that’s why their C-section rate is high. At these times I tell them; well, you have to wait. Maybe he will accept what I have said because I have 26 years of experience and he knows me and my knowledge. Whether she accepts my advice or not is very influential in our communication.”*

Participants reflected that the most important factor in providing optimal care to pregnant woman is to create a sense of trust. One particularly expressed their experience in this area when caring for a woman enduring a high-risk pregnancy: *“You have to do something to gain the patient’s trust. From the beginning of patient arrival, I think the first encounter is very important. If you can gain the trust of patient at the first encounter, you can establish a good relationship with her.”*

## Discussion

The aim of the present study was to explore midwives’ lived experiences of delivering continuous midwife-led intrapartum care. After analyzing the data, “‘Wanting to lead continuous woman-centered care but being unable to” was identified as an overarching theme. Three themes “emphasis on the non-interventional care”, “midwifery-specific focus” and “barriers and challenges of midwifery care” were also identified. Ultimately, participants know what midwifery-led woman-centered care is and why it is valuable. They describe wanting to lead woman-centered care but also how they cannot do so predominantly due to their lack of autonomy and voice in their subordinate positions in the Iranian health professional power hierarchy. This is highly concerning as there have been global calls to scale up the provision of midwifery care globally [24], as midwives have the potential to avert millions of maternal and neonatal deaths worldwide [25]. The roles of midwives can indeed be very valuable and integral in labour management. Thus, midwives and obstetricians should play complementary and not autonomous or antagonists’ roles in labour management, as highlighted by these statements. Workspaces should also purport cordiality and cohesion rather than division.

Midwives within this study expressed their own preference for midwifery-led continuity of care and facilitating physiological birth without unnecessary intervention, with a focus on psychological support. This is encouraging as the results of a study by Attarha et al. (2020) demonstrated that constant presence of midwives along with emotional-psychological support, empathy and understanding promoted improved mental health and quality of life [26]. Indeed, midwifery continuity of care is considered the optimal model of care [11, 12], and may further strengthen relationships [26].

Midwives are responsible for promoting physical, mental, and social health [27]. Working in optimised continuity of care models, midwives can protect such rights to health by avoiding abusive and destructive behaviors; preserving personal privacy; respecting decisions and preferences and through the provision of evidence-based advice [28]. Yet participants here often faced perceived barriers to justice in this pursuit. This is concerning as the protection of rights can reduce hospitalization time, costs, and lead to the prevention of irreparable physical, psychological, and emotional harms [29]. Contrariwise, statements referring to women and birthing people not listening to the advice of midwives call for reflexivity on the part of the midwife to avoid bias and personal aversion to Caesarean Sections and a promotion of ‘normal birth’ at all costs by inadvertently transferring such biases along to perinatal service users.

One major barrier to the provision of midwifery care outlined by participants was that few attend antenatal education and subsequently are unprepared for physiological birth. Elsewhere, the results of a study by Ebert et al. (2014) showed that although some wanted to be decisive and involved in their pregnancy care, they did not receive sufficient information or proper physical and emotional support from midwives. They therefore were reliant upon the instructions or recommendations of specialists [30]. Yet higher awareness in relation to the progress of labour and use of analgesia during labour can enable those experiencing high risk pregnancies in particular to better engage with midwives and the planning of their care [31]. Indeed, in a study by Gibbins and Thomson, all participants were similarly more likely to participate actively in postpartum decision making when receiving information during pregnancy care with midwifery support [32]. Thus, further research is needed to identify why uptake of antenatal education in Iran is poor along with the development of midwife-led programs.

Another challenge in the provision of midwifery care was a perceived lack of midwives’ value and role in decision making. Nevertheless, as midwives are trained to play an integral part in the provision of perinatal care, their professional abilities and competencies should be recognised, and their autonomy respected [33, 34]. Decreased autonomy leads to loss of self-confidence, decreased job satisfaction, and burnout in midwives [35]. Yet as is the case elsewhere [36], midwife participants here report being pressured by obstetricians to expedite labour and follow the medical model of intervention. Whilst the philosophy of midwifery and obstetrics may be at odds, ongoing dysfunctional communication between midwives and obstetricians were seen by participants as significant barriers to the establishment of effective communication between obstetricians and midwives, as they are elsewhere [37]. As such, initiatives aiming to promote mutual professional respect and collegiality would be welcomed in pursuit of better births.

Participants in this study overwhelmingly asserted that the majority of births were attended by obstetricians, with midwives not being given priority as the lead practitioner during low-risk physiological births. It was suggested by participants that the reason for this was at times related to finance and lack of trust. This is concerning as effective professional communication, trust and relationships between midwives and obstetricians can lead to increased patient participation, cooperation, and satisfaction [38]. The lived experiences presented clearly do not highlight what should be happening in practice. Indeed, going forward, it will be important for midwives to work as part of a well-respected multidisciplinary team [39], particularly in cases relating to high-risk pregnancies [40]. As with our findings, the results of a study by Fornman et al. (2019) showed that midwives felt less important than other members of the healthcare team and that this discouraged and unmotivated them making them feel undervalued and absent in the decision-making process [41]. This contradicts global calls to have midwives’ autonomy respected and their involvement in decision-making valued at every level [12, 25]. It is suggested that increased financial remuneration for midwifery work will also be advantageous in boosting the societal value of midwifery work [42].

Challenging working environments full of stress and fatigue due to high workload, shortage of staff and long working hours can lead to medical error, low quality care and poorer outcomes [43, 44]. This is concerning because as is the case elsewhere [35], participants here also experienced increased workload, high stress levels, and reduced self-confidence. Whilst the solutions to these challenges may be complex, the findings of Berg’s study show that midwives’ struggle is not necessarily for the legitimacy of their profession, but for the protection of rights during pregnancy and childbirth [45]. Thus increasing staff support along with the value, recruitment, and retention of midwives will be key in optimizing how midwives overcome such struggles in future [12, 46]. In this pursuit, the professional duties, value, and scope of midwives may be usefully highlighted to healthcare system policymakers [47, 48].

The need to form a triangle of trust in care was commonly mentioned by midwives participating in this study. Midwives should provide care with a balanced view based on evidence and knowledge [49]. In promoting trust, the midwife is entrusted to support those pregnant in making informed decisions [50]. Such trust is considered essential in this context [51], yet our findings point to a need for trusting relationships to be enhanced in Iran. Furthermore, in our study, as is the case elsewhere [52], participants reported their personal feeling that obstetricians did not trust midwives, that midwives were underestimated by obstetricians, and that this sometimes leads to delays in diagnosis and treatment. The reasons for such feelings of distrust must be explored and addressed in future programs of research involving all parties to avoid such outcomes in future.

We note that some participant statements may be disputed, or not universally applicable (e.g., the timings presented in relation to the active phase of labour). Some other perceptions may also be exceptions rather than the norm and may be uncomfortable for some as they connote animosity rather than complementariness in the roles of obstetricians and midwives in labour management. Yet in this context, cordiality between obstetricians and midwives should always be the goal and future research could usefully provide evidence to support this.

### Strengths and Limitations

Due to the nature of qualitative research, our study is limited in having included only a small number of participants in a unique Iranian cultural context. Therefore, our findings cannot be generalized. Nevertheless, we have been able to interpret the lived experiences of 10 midwives working as midwives in Iran. Such voices offer important insights into some of the barriers and challenges of midwives in providing optimal care. Our findings are corroborated by those of previous studies. Yet as our study was exploratory in nature, our findings are not intended to be generalizable [53]. Researcher triangulation has further increased the credibility and validity of research findings.

## Conclusion and policy implications

This study has explored midwives’ lived experiences of delivering continuous midwife-led care which is ‘woman-centered’ during labour and birth. For a counter perspective, this study could usefully be repeated in the same context with a cohort of obstetricians. Ultimately, midwives acknowledged that they knew how to and wanted to provide continuous midwife-led care but were unable to. Perceived barriers included lack of familiarity with and knowledge in relation to childbirth, the insignificant role of midwives in decision making, obstetrician utilitarianism, high workloads along with work-related stress argument-driven communication between midwives and obstetricians and an absence of a ‘triangle of trust’ in care. Future research strategies could usefully focus on the upscaling of midwifery in Iran using continuity of care models, highlighting the value of midwives, identifying why uptake of antenatal education in Iran is poor and the development of evidence based, user friendly and midwife-led programs of care and education. Initiatives aiming to promote mutual professional respect, trust and collegiality and increased remuneration for midwifery work would be also welcomed in pursuit of reducing maternal and infant mortality in Iran.

## Data Availability

The datasets generated and analyzed during the current study are not publicly available due the confidentiality of their information but are available from the corresponding author on reasonable request.
